# An eEF1A1 truncation encoded by PTI-1 exerts its oncogenic effect inside the nucleus

**DOI:** 10.1186/1475-2867-14-17

**Published:** 2014-02-26

**Authors:** Louise D Dahl, Thomas J Corydon, Liina Ränkel, Karen Margrethe Nielsen, Ernst-Martin Füchtbauer, Charlotte R Knudsen

**Affiliations:** 1 Department of Molecular Biology and Genetics, Aarhus University, Gustav Wieds Vej 10C, 8000 Aarhus C, Denmark; 2 Department of Biomedicine, Aarhus University, Aarhus C, Denmark

**Keywords:** *PTI-1*, Oncogene, Nucleus, Localization, UTRs, Translation elongation factor

## Abstract

**Background:**

The oncogene *PTI-1* was originally isolated from a prostate cancer cell line by its capability to transform rat fibroblasts. The PTI-1 mRNA has a very eccentric structure as the 5′UTR is similar to prokaryotic 23S rRNA, while the major open reading frame and the 3′UTR corresponds to a part of the mRNA encoding human translation elongation factor eEF1A1. Thus, the largest open reading frame encodes a truncated version of eEF1A1 lacking the first 67 amino acids, while having three unique N-terminal amino acids. Previously, the UTRs were shown to be a prerequisite for the transforming capacity of the PTI-1 transcript. In this study, we have investigated the possible role of the UTRs in regulating protein expression and localization.

**Methods:**

The protein expression profiles of a number of PTI-1 mRNA variants were studied *in vitro* and *in vivo*. Furthermore, the oncogenic potentials of the same PTI-1 mRNAs were determined by monitoring the capacities of stably transfected cells expressing these mRNAs to induce tumors in nude mice and form foci in cell culture. Finally, the cellular localizations of PTI-1 proteins expressed from these mRNAs were determined by fluorescence microscopy.

**Results:**

The PTI-1 mRNA was found to give rise to multiple protein products that potentially originate from translation initiation at downstream, inframe AUGs within the major open reading frame. At least one of the truncated protein variants was also found to be oncogenic. However, the UTRs did not appear to influence the amount and identities of these truncated protein products. In contrast, our localization studies showed that the UTRs of the transcript promote a nuclear localization of the encoded protein(s).

**Conclusions:**

Translation of the PTI-1 mRNA results in multiple protein products of which (a) truncated variant(s) may play a predominant role during cellular transformation. The PTI-1 UTRs did not seem to play a role in translation regulation, but appeared to contribute to a nuclear localization of the PTI-1 protein(s). This indicates that the PTI-1 protein(s) exert(s) its/their oncogenic function inside the nucleus.

## Background

Prostate tumour inducing gene 1 (*PTI-1*) encodes a variant of eukaryotic elongation factor 1A1 (eEF1A1). The canonical role of eEF1A is to deliver aminoacylated-tRNA to the mRNA-programmed ribosome during translation. In addition, a number of alternative functions in e.g. cytoskeletal organization and signaling pathways have been reported [1].

**Figure 1:**
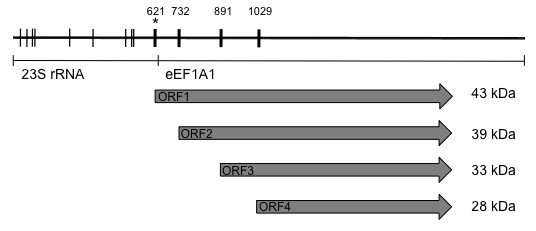
**Schematic overview of the PTI-1 mRNA.** The parts with similarity to 23S rRNA and eEF1A1 mRNA are indicated. Upstream AUGs are marked with thin, vertical lines, while the AUG initiating the longest ORF, ORF1, as well as the first three downstream, inframe AUGs are marked with bold, vertical lines. The first positions of the ORF start codons are numbered according to the full-length PTI-1 mRNA (NCBI accession number L41498). The corresponding ORFs are indicated as grey arrows and the calculated molecular weights resulting upon translation of these ORFs are provided to the right of the arrows. The AUG subject to mutation in the different PTI-1-FL-ΔATG constructs is marked with an asterisk.

The composition of the PTI-1 mRNA (Figure 1) is unusual as its 5′UTR (620 bp) is homologous to 23S ribosomal RNA from *Mycoplasma*, while the 3′UTR (289 bp) is a truncation of the *eEF1A1* 3′UTR [2]. The major open reading frame (ORF1; 1197 bp) encodes a truncated version of eEF1A1 lacking the first 67 amino acids, while presenting three unique N-terminal amino acids. Otherwise, the sequences of the PTI-1 and eEF1A1 proteins are identical [3].

The exact identity of the protein encoded by the PTI-1 mRNA is uncertain despite of the obvious coding potential of ORF1. The start codon flanking ORF1 is found in a suboptimal Kozak context lacking the G^+4^. Furthermore, it is preceded by nine ATG codons in the 5′UTR (Figure 1). This makes high-level expression of the full-length PTI-1 protein unlikely as previously confirmed [3] and opens the possibility that truncated protein products encoded by the mRNA might also be of possible importance during cellular transformation.

*PTI-1* was initially discovered in a prostate cancer cell line, LNCaP, during a screen for new oncogenes [4]. The oncogenic phenotype of the isolated *PTI-1* was confirmed by its ability of rendering stably transfected CREF-Trans 6 cells tumorigenic. Later, downregulation of PTI-1 mRNA levels was shown to reduce growth and induce apoptosis in two other prostate cancer cell lines DU145 and PC3 [5]. The mechanism by which the *PTI-1* gene exerts its oncogenicity is unknown. It has previously been speculated that the oncogenic properties of the PTI-1 protein might result from interference with one or more of the functions of the strongly related eEF1A [6]. Notably, the mammalian isoform of eEF1A specific to brain, heart and muscle cells, eEF1A2, has been shown to be able to transform mammalian cells and to be overexpressed in a number of tumour tissues from e.g. ovary, breast and lung [7].

Remarkably, previous studies demonstrated that only the full-length PTI-1 mRNA is oncogenic, while expression of neither the 5′UTR alone, ORF1 alone, nor the 5′UTR and ORF1 expressed together as separate constructs caused the cells to be tumorigenic [8]. However, the explanation for the essential role played by the PTI-1 UTR regions in transformation remained unknown.

The UTR regions may contain various cis-acting regulatory sequence elements with an impact on mRNA stability, localization and translation [9]. Genes associated with cell proliferation such as oncogenes and tumour suppressor genes tend to present atypically long and complex 5′UTRs that often contain these regulatory elements [10]. In addition, upstream ORFs have been demonstrated to have regulatory potential [11].

In this study, the potential role of the UTRs of the PTI-1 mRNA in regulating translation and protein localization has been investigated in order to understand the essential contribution of these regions of the mRNA to cellular transformation.

## Results

### Protein expression analysis

**Figure 2:**
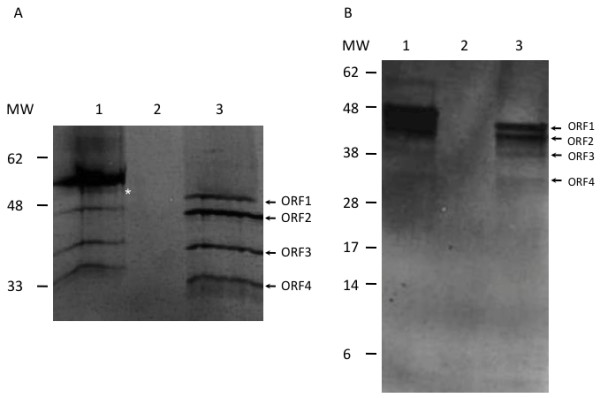
***In vitro *transcription/translation of protein from the *PTI-1 *gene.** Protein products are visualized by gel electrophoresis followed by autoradiography. **A**. Analysis on a 12% polyacrylamide gel. **B**. Analysis on a precast 12% BIS TRIS gel run in MES buffer. Lanes 1: positive control encoding *Xenopus* eEF1A; Lanes 2: negative control without DNA template; Lanes 3: transcription/translation from the pZeoSV2-PTI-1-FL construct. Arrows indicate the bands with potential correspondance to protein products resulting upon translation of ORFs 1, 2, 3 and 4 shown in Figure 1. The weak band in lane 1 marked by an asterisk may correspond to the product resulting upon translation from the second in-frame AUG of the *Xenopus* eEF1A template.

The protein expression profile of full-length PTI-1 mRNA (PTI-1-FL) was initially analyzed using an *in vitro* transcription/translation kit. The resulting products were analysed on two types of gels to allow a clear separation of bands in the area from 25–50 kDa (Figure 2A) and to reveal potential products smaller than 25 kDa (Figure 2B). Several protein products resulted from both the positive *Xenopus* eEF1A control (Figure 2A and B, lane 1) and the full-length PTI-1 template (Figure 2A and B, lane 3). No bands were observed for the negative control without DNA (Figure 2A and B, lane 2). The presence of multiple bands could be due to degradation of mRNA/protein and/or it could be due to translation initiation occurring from multiple AUG codons. No ORFs unrelated to ORF1 (Figure 1) exist in the PTI-1-FL mRNA that could give rise to bands of the observed sizes. However, a number of down-stream, in-frame AUGs exists within ORF1 that could potentially give rise to bands of the observed sizes (marked by bold, vertical lines in Figure 1).

The upper band in the *Xenopus* eEF1A contol corresponds to a protein product of approximately 50 kDa as expected upon initiation from the canonical start codon. Right below the upper band, a faint band is observed, which might represent a protein product of approx. 45 kDa initiated at the second, in-frame AUG codon (marked by an asterisk in Figure 2A). The third band potentially represents a 39-kDa protein product initiated at the third in-frame AUG codon in the *Xenopus* eEF1A template. This band is shared by the PTI-1-FL template (Figure 2A, lane 3) and could represent a protein product corresponding to ORF2 of the PTI-1-FL template (Figure 1). Above the common band of 39 kDa, a product of approximately 43 kDa is observed for the PTI-1-FL template, which fit with the size expected upon translation from ORF1. The two bands migrating faster than the 39 kDa band observed for both the *Xenopus* eEF1A and the PTI-1-FL template, could potentially represent protein products of 33 and 28 kDa originating from translation initiations from successive downstream, in-frame AUGs, which are all conserved between the two sequences (corresponding to ORF3 and ORF4 of the PTI-1 mRNA, Figure 1). No proteins of lower molecular weight could be clearly distinguished (Figure 2B).

The intensities of products potentially resulting from initiations at downstream AUGs in the full-length PTI-1 mRNA were similar to that of the largest product indicating a possible role of these smaller proteins in cellular transformation. Remarkably, the expression that is likely to originate from ORF2 appeared to be higher than that observed from ORF1 (Figure 2, lane 3).

**Figure 3:**
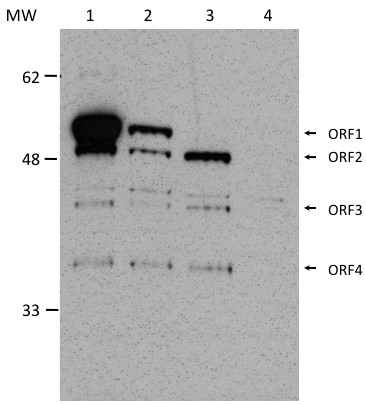
**Transient expression of V5-tagged PTI-1 *in vivo*.** NIH 3 T3 cells were transiently transfected with the following constructs: pcDNA3.1-PTI-1-ORF1-V5 (lane 1), pcDNA3.1-PTI-1-FL-V5 (lane 2), pcDNA3.1-PTI-1-FL-ΔATG-V5 (lane 3) or an empty pcDNA3.1 vector (lane 4). Cells were harvested and lysed approximately 48 hours post transfection. 40 μg of total protein lysate was loaded in all lanes. Expression was analyzed by Western blotting using an anti-V5-antibody. The presence of the V5 tag increases the molecular weight of the expression product by 2.6 kDa. Arrows indicate the bands with potential correspondance to protein products resulting upon translation of ORFs 1, 2, 3 and 4 shown in Figure 1. A background band of unknown origin appears above the ORF3 band in all lanes and serves as a loading control.

*In vitro* transcription/translation systems are known to give rise to internal initiation of translation that does not take place *in vivo*[12,13]. This inherent flaw of the system might give rise to protein products whose translation *in vivo* would be hindered by the 5′UTR of the PTI-1 mRNA. Thus, *in vivo* analysis of transient protein expression from constructs containing the sequence encoding a V5-tag situated immediately before the stop codon flanking ORF1 was conducted. This allowed the detection of full-length protein products as well as products initiated from downstream, inframe AUGs. In this analysis, two additional constructs were included: a construct giving rise to an mRNA containing only ORF1 without UTRs (pcDNA3.1-PTI-1-ORF1-V5) and a full-length construct with a mutation of the startcodon of ORF1 (pcDNA3.1-PTI-1-FL-ΔATG-V5). The first construct allowed an evaluation of the possible regulatory effect of the 5′UTR on translation, while the second construct was included to substantiate our theory that downstream in-frame AUGs might be recognized during translation initiation. The sizes of the proteins expressed *in vivo* in transfected NIH 3 T3 cells (Figure 3) were very similar to those of the products observed *in vitro* (Figure 2). Again, four specific protein products could be detected, likely representing initiation from each of the first four in-frame AUGs in ORF1. No translation products could be detected for any of the succeeding, potential downstream ORFs. Notably, the upper band obtained from the pcDNA3.1-PTI-1-FL-ΔATG-V5 construct (Figure 3, lane 3) migrated to the same position as the second band in lanes 1 and 2 indicating that the latter bands most likely stem from translation of ORF2 (Figure 1). Remarkably, the products obtained using the ORF1-only template and the full-length template were the same i.e. the 5′UTR did not appear to change the identity of the proteins expressed, but the intensities of the two upper bands (ORF1 and ORF2) were markedly reduced in the cells transfected with pcDNA3.1-PTI-1-FL-V5. In general, initiation from downstream AUGs seemed reduced *in vivo*. However, potential expression from ORF2 still appeared reasonably high and might give rise to a protein of relevance as an oncoprotein.

### Oncogenicity assays

The multiple potential expression products observed upon expression *in vitro* (Figure 2) and *in vivo* (Figure 3) indicated that the pattern of expression from the *PTI-1* gene was more complex than previously anticipated. Thus, we found it important to evaluate the transforming potential of protein products resulting upon translation from downstream, inframe AUGs.

The oncogenic potentials of variants of the *PTI-1* gene were analyzed using the constructs pZeoSV2-PTI-1-ORF1, pZeoSV2-PTI-1-FL and pZeoSV2-PTI-1-FL-ΔATG expressing PTI-1 mRNAs comprising ORF1 only, the full-length mRNA or the full-length mRNA with a point-mutation in the start codon of ORF1, respectively. The constructs were stably transfected into NIH 3 T3 cells and analysed for their ability to induce tumor formation in nude mice and/or to form foci in culture. Cells stably transfected with an empty pZeoSV2 vector were used as a negative control. Four sets of stable cell lines were established and analyzed independently. The first and second sets of stable cell lines were evaluated for their abilities to induce tumors in nude mice, while the foci-forming capacities of the second, third and fouth sets were determined i.e. the transforming potential of the second set of stable cell lines was analyzed using two different approaches. Expression of the integrated constructs was confirmed by RT-PCR (data not shown). The cells were tested free of *Mycoplasma* contamination (data not shown). Each 36-mm well was estimated to give rise to approximately 500 individually transfected clones. No clonal isolation was performed, and the oncogenicity assays were conducted on pools of clones.

**Figure 4:**
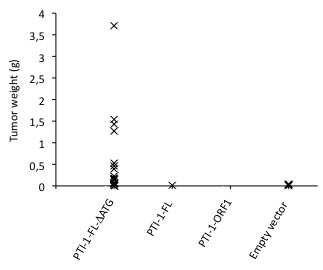
**Tumour development 35 days after injection of nude mice with NIH 3 T3 cells stably transfected with pZeoSV2 constructs expressing variants of the PTI-1 mRNA.** Each tumour is represented as a cross. A total of 20 injections were made for each construct. The number of tumours arisen after injection with cells expressing the various constructs were as follows: pZeoSV2-PTI-1-ORF1: 0, pZeoSV2-PTI-1-FL: 1; pZeoSV2-PTI-1-FL-ΔATG: 18 and empty pZeoSV2 vector: 4.

The abilities of the stably transfected cell lines to induce tumours in nude mice upon subcutaneous injection were tested with the first two sets of independently established cell lines. The first set of cell lines was injected in quadruple (data not shown), whereafter the experiment was repeated on a larger scale for the second set of cell lines. Similar results were obtained in the two sets of experiments. For the second set of transfections, 20 injections were made into immunodeficient NCr nude mice for each stable cell line. 35 days after injection, the mice injected with the PTI-1-FL-ΔATG-transfected cells displayed 18 visible tumours. Amongst the other mice, a total of only six small tumours were observed. The PTI-1-FL-ΔATG-expressing cell line clearly gave rise to more and larger tumours than any of the other cell lines (Figure 4).

**Figure 5:**
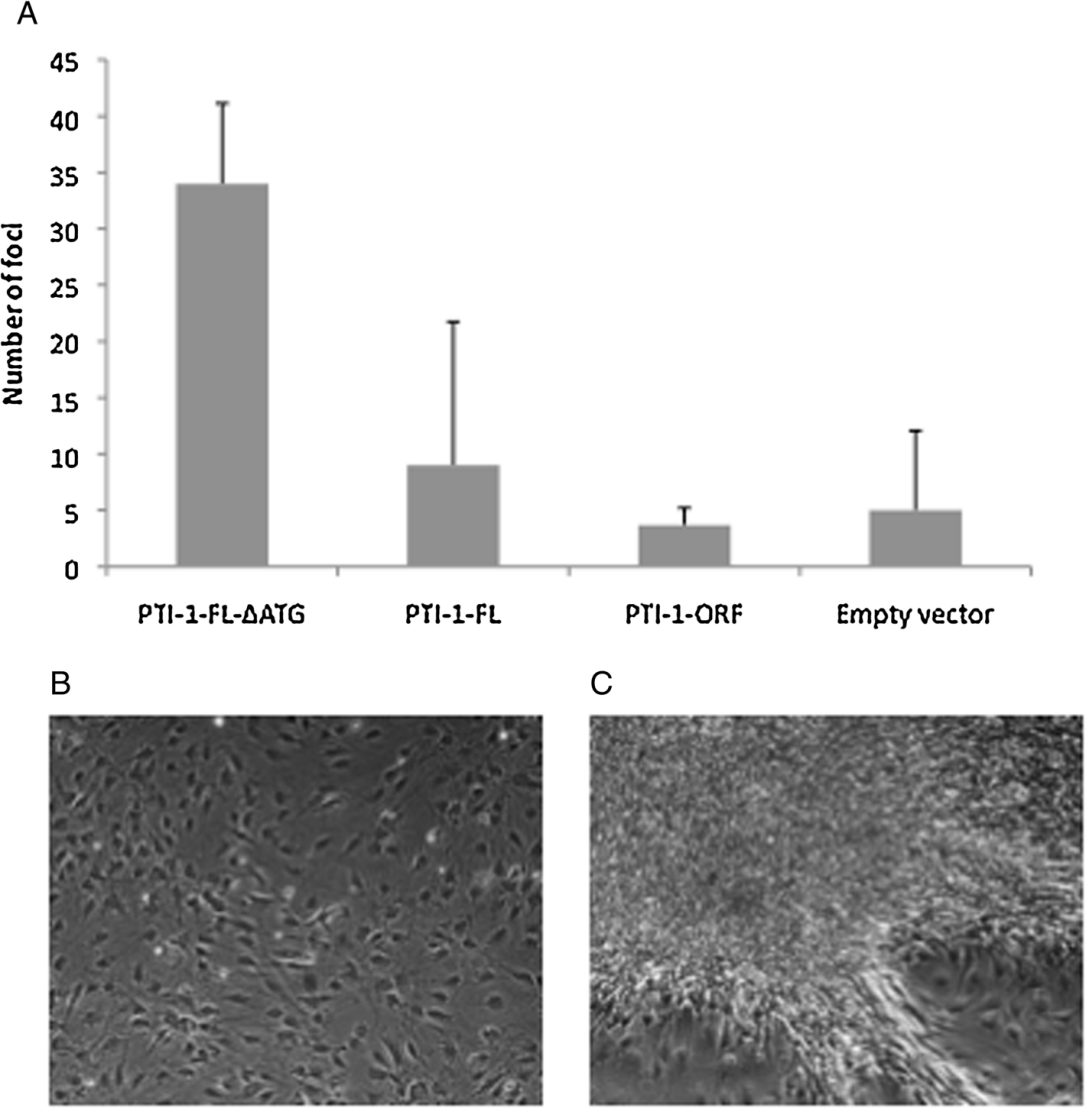
**Foci formation at 5% serum.** NIH 3 T3 cells were stably transfected with the indicated constructs. Three sets of cell lines were independently established and analyzed for each construct. **(A)** shows the number of foci counted in the various cell lines, while **(B)** and **(C)** depict representative examples of background growth and foci growth, respectively. In A, standard deviations are shown. The construct expressing the full-length PTI-1 mRNA with a mutation in the start codon of ORF1 is clearly transforming (a comparison of the number of foci obtained for the pZeoSV2-PTI-1-FL-ΔATG and empty pZeoSV2 in a Student's *t*-test results in a *P* value of 0.024).

All of the cell lines established during the second, third and fourth sets of stable transfections were tested for their foci forming potential *in vitro*. The experiments were conducted in cells that had been propagated at subconfluency for 12 days after ended zeocin selection. Subsequently, the cells were seeded subconfluently and incubated for three weeks in the presence of 5% serum, whereupon foci were scored (Figure 5). In correspondance with the mouse experiments (Figure 4), the transforming capacity of the PTI-1-FL-ΔATG expressing cell line was statistically significant (Student's *t*-test, *P* < 0.03 upon comparison with the number of foci obtained with the empty vector).

### Protein localisation

**Figure 6:**
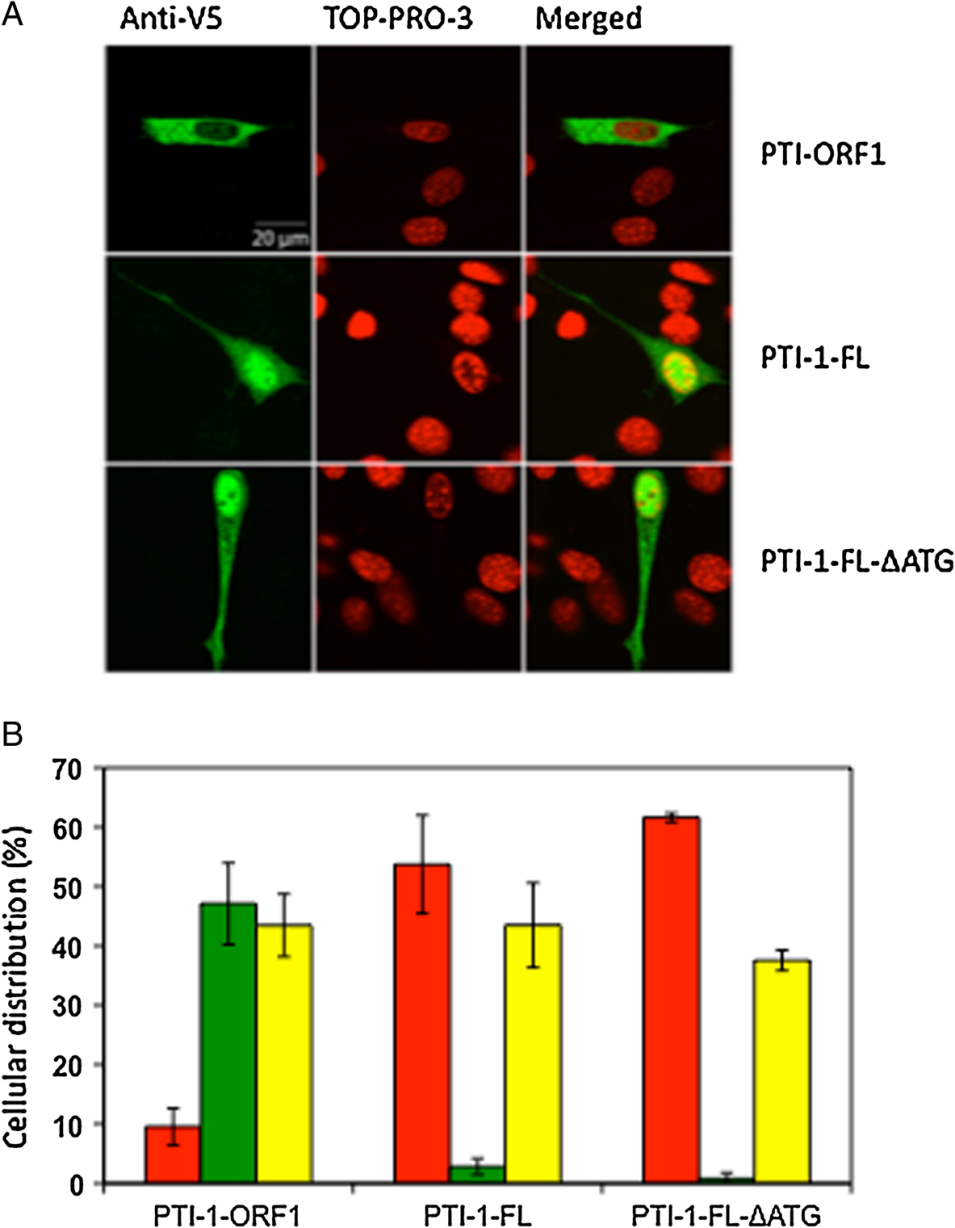
**Intracellular localization of PTI-1 protein.** V5-tagged PTI-1 protein was transiently expressed in NIH 3 T3 cells from the constructs pcDNA3.1-PTI-1-ORF1, pcDNA3.1-PTI-FL and pcDNA3.1-PTI-1-FL-ΔATG. The cells were fixed and stained with mouse anti-V5 antibody and Alexa-fluor® 488 goat anti-mouse antibody (green) and the DNA stain TO-PRO-3 (red). **(A)** Images recorded by CLSM using a x100 oil-immersion objective showing the localization of V5-tagged PTI-1 protein and nuclear DNA. **(B)** Scoring of the cellular distribution of PTI-1 protein. The transiently transfected cells were observed under a fluorescent microscope. The assay was repeated three times with three independent transfections with each construct. Over 100 cells were scored each time for each construct transfection. The cells were assigned to one of three categories: mainly nuclear localization (red bars), mainly cytoplasmatic localization (green bars) or even distribution between the nucleus and the cytoplasm (yellow bars). Standard deviations are shown.

As the 5′UTR seemed to be of only minor importance for the PTI-1 protein expression pattern, the effect of this mRNA region on protein localization was investigated. Transiently expressed PTI-1 protein was fluorescently stained via a C-terminal V5-tag. During transfections, the amount of DNA was kept relatively low to avoid potential overload of the internal translocation systems as well as passive diffusion of protein via nuclear pores. CLSM (Figure 6A) revealed that PTI-1 expressed from pcDNA3.1-PTI-1-ORF1-V5 containing no UTR regions was primarily localized in the cytoplasm, whereas PTI-1 expressed from cells tranfected with full-length constructs, pcDNA3.1-PTI-1-FL-V5 or pcDNA3.1-PTI-1-FL-ΔATG-V5, was found mainly in the nucleus. For statistical purposes, the assay was repeated three times and the cells were counted in a fluorescence microscope and scored in three catagories: mainly nuclear localization, mainly cytoplasmic localization or even distribution between the two compartments. Over 100 cells were counted for each construct. The result (Figure 6B) confirms the observations made during confocal microscopy and strongly suggests that the UTRs of the PTI-1 mRNAs examined are critical for the nuclear localization of the corresponding PTI-1 protein.

## Discussion

### Protein expression from the full-length PTI-1 transcript

The nature of the protein(s) encoded by the PTI-1 gene is so far unknown. The detection of the largest possible protein that can result after translation of the PTI-1 mRNA (corresponding to ORF1) has been attempted using eEF1A-specific antibodies. The results appear controversial, but may depend on cell type [3,5,14]. A theoretical study suggests the expression of a relaxin homolog from an uORF with potential influence on prostate cancer progression [15]. However, no experimental studies have so far addressed the possible expression of truncated proteins from the PTI-1 mRNA. Thus, the nature of the protein encoded by the PTI-1 gene has stayed uncertain.

In this study, the expression profile of the *PTI-1* gene was therefore analyzed *in vitro* and *in vivo* to determine the size(s) of any protein(s) expressed from the full-length gene. The result of the *in vitro* analysis indicated the existence of a complex pattern of translation initiation from downstream, inframe AUG codons (Figure 2).

A similar expression pattern has been observed by others (Figure 4 in [2]). However, these authors ascribed the protein products of lower molecular weight to mRNA/protein degradation or artificial initiation at down-stream AUGs, and thus considered these proteins unimportant. Yet, our subsequent examination of the expression profile of the full-length transcript *in vivo* substantiated the interpretation of different, faster migrating protein products as the results of translation of downstream, in-frame ORFs as the exact same expression pattern was observed (compare Figures 2 and 3). Importantly, the largest protein product resulting after translation of the PTI-1-FL-ΔATG construct migrated to the same position as the second band observed upon expression from the corresponding, unmutated construct indicating that this band may indeed be the result of translation of ORF2. Thus, the original assignment of the first ATG in ORF1 as the start codon of the onco-protein may be an oversimplification [2]. It is just as likely that one or more of the shorter products may represent the oncogenic factor(s). This notion is emphasized by the observed transforming capacity of the PTI-1-FL-ΔATG construct (Figures 4 and 5). Notably, removal of the start codon flanking ORF1 by site-directed mutagenesis increased translation from ORF2 (Figure 3) correlating with a potential role for this eEF1A1 truncation in the transforming capacity of the PTI-1-FL-ΔATG construct.

Similar qualitative expression patterns of eEF1A1 truncations were observed in NIH 3 T3 cells transfected with the PTI-1-ORF1, PTI-1-FL and PTI-1-FL-ΔATG constructs (with the absence of the uppermost band in the lysates of the PTI-1-FL-ΔATG transfected cells) (Figure 3). No protein product seemed expressed in higher amount from the PTI-1-FL construct as compared to the PTI-1-ORF1 construct. This demonstrates that the 5′UTR does not cause an altered expression pattern with a higher level of an oncogenic variant. The explanation for the requirement of the UTRs for the oncogenicity of the PTI-1 gene should therefore be sought elsewhere.

The expression of a small peptide (approx. 4.6 kDa) from a uORF as suggested in a computational analysis of the expression potential of the PTI-1 mRNA [15] could not be confirmed *in vitro* (Figure 2B), but cannot be excluded to occur *in vivo*.

### The oncogenic potential of the PTI-1 gene

The oncogenic potential of the *PTI-1* gene was assessed via the generation and analysis of stable cell lines expressing different variants of the PTI-1 mRNA. Each cell line was established independently four times in order to rule out the risk that observed, tumorigenic effects were caused by a single clone with construct-independent, malign characteristics. A similar pattern of oncogenic potentials was observed for each set of cell lines.

As expected, PTI-1-ORF1 expression did not give rise to tumors or significant levels of foci [8]. The anticipated transforming property of the PTI-1-FL construct [8] was not convincingly demonstrated under the conditions applied in this study in accordance with the previously reported inefficiency of NIH 3 T3 cells as reporters of this activity [4]. In contrast, the PTI-1-FL-ΔATG expressing cell line gave rise to multiple tumours and foci and may be a stronger oncogenic construct than PTI-1-FL. This indicates that the responsible oncoprotein is encoded by an ORF, which is shorter than the originally anticipated ORF1 [2]. Assuming this was the case, removal of the start codon initiating ORF1 would facilitate translation initiation at downstream AUG codons and give rise to increased levels of the oncoprotein, which might thereby pass the threshold required for transformation to occur (Figure 3; compare the intensities of the potential ORF2 products in lanes 2 and 3). The construct devoid of the UTR regions expresses a similar amount of ORF2 product (Figure 3, lanes 1 and 3 display the same intensities of the potential ORF2 product) without being transforming. Thus, our oncogenicity experiments support previous experiments showing that the PTI-1 UTRs are required for the transforming capacity of *PTI-1*[8]. Since the mutation of the ORF1 start-codon does not affect the oncogenic properties of full-length *PTI-1* mRNA negatively, a more truncated version of eEF1A1 must also be oncogenic. Additional experiments are required to pinpoint, which eEF1A1 truncations are responsible for eliciting the transformed phenotype.

### Cellular localization of the PTI-1 protein

In eukaryotic cells, a large proportion of specific mRNA species exhibit a distinct localization within the cell as a mechanism of providing for localized synthesis of the encoded protein close to its site of action [16]. The PTI-1 proteins expressed from the UTR-containing constructs displayed a predominant nuclear localization, while the mRNA containing only ORF1 gave rise to a protein, which was primarily located in the cytoplasm (Figure 6).

The fact that the pattern of subcellular localization was independent of the amino acid sequence of PTI-1 rules out a post-translational mechanism of localization based on e.g. the presence of a nuclear localization signal. A more plausible explanation could be that the mRNA is localized to the perinuclear area and that subsequent translation of the protein close to the nucleus facilitates its nuclear import. The nuclear localization of a number of proteins including among others c-fos, c-myc and metallothionein-1 (MT-1) appear to be depending on zipcodes in the 3′UTRs of mRNAs, which are consequently anchored in the perinuclear region via interactions with trans-acting factors [17-19]. Upon translation, the immediate vicinity of its final destination facilitates nuclear import of the resulting protein.

A similar scenario of perinuclear mRNA anchoring followed by protein import to the nucleus can be envisaged for PTI-1. The majority of known zipcodes are found in the 3′UTR, but examples of a 5′UTR localization exist as well e.g. in the *Drosophila gurken* mRNA [20]. Either of these possible localizations of zip codes remains open in the case of PTI-1. The 3′UTR is shared between PTI-1 and eEF1A1 mRNA, while the 5′UTR is unique to PTI-1 and could therefore be held responsible for the nuclear targeting of PTI-1 products. In contrast, the presence of an independently functioning zip code in the ORF regions as observed in the yeast *Ash1* transcript [21] can be excluded.

### Oncogenic effects of the PTI-1 protein in the nucleus

The results obtained in this study indicate that the nuclear localization of proteins expressed from UTR-containing PTI-1 transcripts is a prerequisite for them to exert their oncogenic properties. How does the nuclear presence of one or more eEF1A1 truncations cause transformation?

eEF1A is generally considered a cytoplasmatic protein [22,23] in accordance with its primary role in translation. A number of studies, however, report the nuclear presence of eEF1A in relation to e.g. apoptosis [24], cell proliferation [25] and cancer [26]. It is tempting to suggest that the abnormal presence of truncated forms of eEF1A in the nucleus may interfere with different nuclear functions of eEF1A thereby leading to dysregulation of these with the potential of causing cancer. The most relevant, potential roles of eEF1A1 in the nucleus that could be affected by the company of PTI-1 are described in the following.

The nuclear presence of eEF1A may be a normal feature of proliferating cells. It has been observed that eEF1A is recruited to the nucleus upon mitogen stimulation and assist the zinc finger protein ZPR1 in driving cell cycle progression [25,27]. The promiscuous presence of PTI-1 in the nucleus could potentially invoke a misregulation of this pathway leading to mitogen-independent proliferation.

eEF1A1 and −2 have both been reported to interact with sphingosine kinases 1 and 2 (SK1, SK2) and cause their activation [28,29]. The canonical role of SK1 is to catalyse the formation of sphingosine-1-phosphate (S1P) from a sphingolipid precursor. In contrast to the precursor, S1P promotes cellular division and inhibit apoptosis. While SK1 appears to be primarily cytosolic, SK2 shuttles between the nucleus and the cytoplasm, [30]. SK2 has been shown to play a distinct role from SK1 and to inhibit growth and induce apoptosis [31-33]. The nuclear function of SK2 is unknown and might involve S1P independent pathways.

Akt is a threonine/serine kinase that regulates the activities of numerous targets related to cell survival and proliferation. Akt itself is activated via phosphorylation by phosphoinositide-dependent protein kinase 1 (PDK1). Phosphorylation of Akt facilitates its nuclear translocation and retention [34]. Yet, the role of pAkt in the nucleus is not clear and may be related to its well-known pro-proliferative/anti-apoptotic function or to newly discovered pro-apoptotic pathways [35,36]. eEF1A has been found to interact with pAkt [37,38]. Thus, PTI-1 could potentially interact with nuclear pAkt and interfere with its signaling activities.

## Conclusions

The PTI-1 oncogene potentially encodes a number of shortened variants of translation elongation factor eEF1A1. We found that the untranslated regions (UTRs), which are essential for transformation, do not influence on protein expression, but plays a critical role during nuclear localization of the encoded oncoprotein product(s), which may be shorter than previously predicted. Several eEF1A interaction partners are found in the nucleus, where they regulate cell proliferation and survival. The nuclear localization of eEF1A truncations encoded by the PTI-1 transcript may interfere with the pathways of the eEF1A interaction partners possibly provoking misregulation of the cell cycle, escape from apoptosis, and neoplastic progression.

## Materials and methods

### DNA constructs

The *PTI-1* DNA was previously obtained from Dr. Fisher, Columbia University, New York and sequenced upon receipt [3]. The donated fragment is identical to the ~ 1.9 kb oncogenic fragment described by Su *et al.*[4] and will be referred to as full-length *PTI-1* throughout the paper. For site-directed mutagenesis, the QuickChange® II Site-Directed Mutagenesis Kit (Stratagene) was employed.

The constructs pZeoSV2-PTI-1-ORF1, pZeoSV2-PTI-1-FL and pZeoSV2-PTI-1-FL-ΔATG were employed in oncogenicity assays as described below. The pZeoSV2 vector (Invitrogen) was chosen for these experiments, since the same vector had previously been used to demonstrate the oncogenicity of *PTI-1*[8]. Furthermore, these vectors were applied for *in vitro* analysis of protein expression profiles (see below). The full-length *PTI-1* gene and the ORF1 of the *PTI-1* gene were cloned into pZeoSV2 resulting in the constructs pZeoSV2-PTI-1-FL and pZeoSV2-PTI-1-ORF1 respectively. The construct pZeoSV2-PTI-1-FL-ΔATG was created by site-directed mutagenesis of pZeoSV2-PTI-1-FL exchanging the T in start codon initiating ORF1 for a C.

The constructs pcDNA3.1-PTI-1-FL-V5, pcDNA3.1-PTI-1-ORF1-V5 and pcDNA3.1-PTI-1-FL-ΔATG-V5 were employed for the analysis of protein expression profiles *in vivo*. The PTI-FL and PTI-1-ORF1 inserts were subcloned from their respective pZeoSV2 constructs into the pcDNA3.1 vector. Next, a sequence encoding a C-terminal V5-tag was inserted in-frame by site-directed mutagenesis thereby creating pcDNA3.1-PTI-1-FL-V5 and pcDNA3.1-PTI-1-ORF1-V5. The construct pcDNA3.1-PTI-1-FL-ΔATG-V5 was generated by mutating the start codon of the pcDNA3.1-PTI-1-FL-V5 vector in a manner identical to the one described for mutagenesis of the startcodon in pZeoSV2-PTI-1-FL.

For all constructs, the correctness of the insertions and the integrity of the constructs were confirmed by sequencing. In addition, the correctness of promotor sequences and polyadenylation sites were confirmed.

### Protein expression analysis

Protein expression analysis *in vitro* was performed using the transcription/translation assay kit, Proteinscript® II T7 kit from Ambion. [^35^S]-methionine was purchased from Perkin Elmer. Transcription reactions were carried out as recommended by the manufacturer. DNA templates were the linearized pZeoSV2-PTI-1-FL and the positive control pTRI-Xef included in the kit. 0.5 μg DNA was used in each reaction. A negative control reaction without DNA was included. The transcription reactions were used as templates in the translation reactions. The positive control reaction was mixed as prescribed by the protocol containing a final methionine concentration of 30 μM of which 0.5 μM were [^35^S]-methionine (1100 Ci/mmol). For the other two reactions, the final methionine concentration was 25 μM of which 1 μM was [^35^S]-methionine (1100 Ci/mmol). 15-μl portions of each of the translation reactions were loaded and run on a 12% SDS polyacrylamide gel or a precast 12% BIS TRIS gel (Bio-Rad). The gel was dried in a gel dryer. An X-ray film (Konica Minolta) was exposed to the dried gel for 2 days.

The *in vivo* protein expression profile of the PTI-1 gene was accessed using the constructs pcDNA3.1-PTI-1-FL-V5, pcDNA3.1-PTI-1-FL-ΔATG-V5, and pcDNA3.1-PTI-1-ORF1-V5. Transient transfection was performed as described for the establishment of stable cell lines (see below) with the exception that circular DNA was employed. Six hours after transfection, the medium was changed. The cells were incubated for approximately 48 hours before they were harvested for analysis. A transfection with pZeoSV2/*lac*Z was always included as a transfection control. A negative control was transfected with an empty pcDNA3.1 vector. The harvested cells were washed in PBS (137 mM NaCl, 2.7 mM KCl, 10 mM Na_2_HPO_4_, 2 mM KH_2_PO_4_), resuspended in 100 μl lysis buffer (150 mM NaCl, 10 mM MgCl_2_, 1 mM DTT, 10% glycerol, 0,5% NP-40, 50 mM Tris/HCl pH 7.5 and complete EDTA-free inhibitor (Roche)), and incubated for one hour on ice. The protein concentration of the lysate was determined using the Bradford assay [39]. The protein samples were run on a 10% SDS polyacrylamide gel and subjected to Western blotting. V5-tagged PTI-1 was detected with a HRP-conjugated rabbit anti-V5 tag polyclonal antibody (Genscript) used in a concentration of 0.5 μg/ml. The blot was developed with the ECL Plus Western blotting detection system (GE Healthcare) and visualized on X-ray film (Konica Minolta).

### Cell culture

NIH 3 T3 cells were purchased from the American Type Culture Collection and their alleged contact inhibited growth was confirmed in a foci-formation assay. The number of colonies arising after transfection of a 36-mm well was estimated in an experiment where selection was performed on a very large growth area omitting the need for splitting the cells during selection.

Cells were grown in DMEM (DMEM + GlutaMAX™-I from GIBCO) containing 10% bovine calf serum (SAFC Biosciences), 100 units penicillin/ml, and 100 μg streptomycin/ml. The cells were split regularly to keep them below 80% confluency at all times.

### Establishment of stable cell lines

The pZeoSV2 constructs were linearized with BspHI and purified by phenol**-**chloroform extraction. NIH 3 T3 cells were seeded in 36-mm wells, 400.000 cells per well. The following day, the cells were transfected with 4 μg DNA using Lipofectamine™ 2000 transfection reagent (Invitrogen). The DNA used for transfection was diluted in OPTI-MEM^®^I (GIBCO) and sterile filtered prior to transfection. Duplicate or triplicate transfections were made with each construct. A transient transfection with pZeoSV2/*lac*Z was always included as a transfection control. Six hours after transfection, the cells were reseeded onto larger surfaces. Two days after transfection, zeocin (Invitrogen) was included in the medium at 0.5 mg/ml. This concentration was chosen based on kill curves as described in the zeocin manual. Plasmocin (Invivogen) was also included in the medium at a concentration of 5 μg/ml to prevent *Mycoplasma* infection. The medium and antibiotics were changed every 3–4 days during selection. The cells were reseeded regularly to avoid incubation at confluency. The selection was considered finished when no more cells remained in the nontransfected control flasks (after 24 days). At that point, zeocin was no longer included in the growth medium of the cells. In contrast, plasmocin was maintained in the medium until the day of injection into mice (see below). Stably transfected cell lines were tested free of *Mycoplasma* by either Hoechst staining or by PCR using the Mycosensor™ PCR Assay kit (Stratagene).

RT-PCR was applied to the stably transfected cell lines to confirm the expression of integrated constructs as follows: mRNA was extracted from one million cells of each stable cell line with the RNeasy® Mini Kit (Qiagen). A DNase cleavage step was included employing RNase-free DNase (Qiagen). The purified RNA was used as a template in an RT-PCR reaction using the OneStep RT-PCR Kit (Qiagen). DNA contamination controls were included in which the reverse transcription step was omitted. The amplification of the PTI-1-ORF1 and empty vector pZeoSV2 transcripts was performed with vector-specific primers RTplasrev (5′-CTAGAAGGCACAGTCGAGGCTG-3′) and RTplasfor (5′-TAGAGAACCCACTGCTTACTGGC-3′). The transcripts from PTI-1-FL and PTI-1-FL-ΔATG were amplified with a primer matching the PTI-1 5′UTR, RT23Srev (5′-CAGCTAGATGCCGCCATTCCAC-3′), combined with the vector-specific primer, RTplasfor.

### Foci forming assays

The experiments were performed on stably transfected NIH 3 T3 cells that had been propagated at subconfluency for 12 days after ended zeocin selection. Then, 400,000 cells of each cell line were seeded subconfluently in a T25 flask in medium containing 10% serum. The following day, the medium was changed to 5% serum and the cells were incubated for three weeks. The medium was changed every 3–4 days. The cells were fixed in icecold methanol and stained with Giemsa stain (Giemsas Azur-Eosin Methylene blue solution from Merck) and colonies were scored.

### Tumorigenicity assay

The established stable cell lines were propagated. On the day of injection, the cells were trypsinized (Trypsin EDTA 170,000 U/l; Lonza), resuspended in medium and counted in a Coulter counter. The cells were washed once in Hank’s balanced salt solution (HBSS; Lonza), resuspended in HBSS and kept on ice until the moment of injection into NCr nude mice (Taconic Europe). Two injections were made on each mouse, one on each flank. Each injection contained 5 million cells. After the injection, the remaining cells were seeded in flasks and proven to be fully viable. Tumor development was followed with a slide gauge using the formula: V = (π/6)×((l + w)/2)^3^ in which V = volume/cm^3^, l = length/cm, w = width/cm. 1 cm^3^ tumor is assumed to weigh 1 g. The mice were sacrificed before the tumor reached a size of 1 cm^3^. The tumors were excised and weighed.

### Protein localization

NIH 3 T3 cells were transiently transfected with pcDNA3.1-PTI-1-FL-V5, pcDNA3.1-PTI-1-ORF1-V5, pcDNA3.1-PTI-1-FL-ΔATG-V5 and the empty pcDNA3.1 vector using the Lipofectamine™ 2000 transfection reagent (Invitrogen). For each transfection, 0.2 μg of the relevant construct was mixed with 3.8 μg of the empty vector. The day after transfection, the cells were reseeded into 9 cm^2^ slideflasks with 200,000 cells per flask. The following day, the cells were fixed and stained as follows: The slides that were used for cell counting were incubated with Hoechst 33258 prior to fixation. The cells were washed with Dulbecco's Phosphate Buffered Saline (DPBS; Lonza) and fixed by incubation in neutral buffered formalin (Lilly’s liquid; VWR) followed by fixation in 70% ethanol at −18°C. The slides were washed in PBS prior to a 1-hour incubation with anti-V5 antibody (Anti-V5 Antibody mouse monoclonal IgG_2a_ from Invitrogen) diluted 1/100 in 50 μl blocking buffer (0.5% BSA, 15 mM NaN_3_). The slides were washed in PBS and incubated for 1 hour with Alexa Fluor® 488 goat anti–mouse IgG antibody (Invitrogen) diluted to 5 μg/ml in 50 μl blocking buffer. The slides were finally washed in PBS. The cells were scored under a fluorescent microscope (Zeiss Axioplan 2 Imaging) using a × 63 objective and Zeiss filter set 10 (Excitation: 450–490 nm, Beam Splitter: 510 nm, Emission 515–565 nm) for visualization of Alexa 488 and Zeiss filter set 34 (Excitation: 390/22 nm, Beam Splitter: 420 nm, Emission: 460/50 nm) for visualization of Hoescht 33258.

The slides to be analyzed by confocal laser scanning microscopy (CLSM) were added 1 μM TO-PRO-3 (Molecular Probes, Invitrogen) in PBS for 5 minutes to stain the DNA and washed twice in PBS as previously described [40]. The slides were added anti-fade mounting medium (VECTASHIELD® Mounting Medium from VECTOR laboratories) and sealed. The cells were analyzed by CLSM (TCS SL, Leica) using a ×100 oil-immersion objective with a numerical aperture of 1.4. Imaging was performed by using a 488 nm line of a multiline argon laser (detection of Alexa-488) and the 633 nm line of a helium-neon laser (detection of TO-PRO-3).

## Abbreviations

CLSM: Confocal laser scanning microscopy; eEF1A1: Eukaryotic elongation factor 1A1; MT-1: Metallothionein-1; ORF: Open reading frame; PCNA: Proliferating cell nuclear antigen; PTI-1: Prostate tumour inducing gene 1; PTI-1-FL: Full-length PTI-1 mRNA; S1P: Sphingosine-1-phosphate; SK: Sphingosine kinase; UTR: Untranslated region.

## Competing interests

The authors declare that they have no competing interest.

## Authors’ contributions

LDD participated in the design of the study, carried out the majority of the expression-, tumorigenicity-, and localization studies and helped to draft the manuscript. TJC participated in the localization studies. LR participated in the expression and localization studies. KMN participated in the expression and tumorigenicity studies. EMF participated in the tumorigenicity studies. CRK conceived of the study, and participated in its design and coordination and drafted the manuscript. All authors read and approved the final manuscript.
